# Gaming in Medical Education: Promise, Pitfalls, and Practice

**DOI:** 10.7759/cureus.114737

**Published:** 2026-08-18

**Authors:** Adalyne Singh, Vijay Rajput

**Affiliations:** 1 Medical Education, Nova Southeastern University Dr. Kiran C. Patel College of Allopathic Medicine, Fort Lauderdale, USA

**Keywords:** educational gaming, gamification in healthcare, higher education medical training, pre-clerkship, problem-based learning (pbl), team-based learning (tbl)

## Abstract

Gaming and gamification have progressively evolved as innovative educational strategies that are increasingly integrated into medical education across the continuum, from pre-clerkship and clinical training to residency and continuing professional development. Advances in educational technologies such as quiz-based platforms, virtual reality (VR), simulation, escape rooms, serious games, and mobile learning applications are increasingly used to promote learner engagement, reinforce knowledge acquisition, and provide psychologically safe opportunities to practice clinical decision-making before caring for real patients.

Current evidence suggests that gaming can improve learner motivation, short-term knowledge retention, procedural competence, and collaborative learning when aligned with educational objectives and supported by deliberate practice, feedback, and structured debriefing. However, the literature also highlights important challenges, including overreliance on extrinsic rewards, superficial learning, inequities in access to technology, and ethical concerns regarding competition and the potential trivialization of patient care. Effective implementation depends on thoughtful instructional design that prioritizes competency-based learning, psychological safety, inclusivity, and reflective practice over entertainment during learning. As medical education continues to evolve, gaming should be viewed not as a replacement for traditional teaching but as a complementary pedagogical strategy that can enhance learning when integrated intentionally within evidence-based educational frameworks and learning goals.

## Introduction and background

Gaming and gamification have moved from novelty to near-mainstream across the continuum of medical education. The growing popularity of these approaches reflects broader shifts in medical education toward active learning, formative assessment, competency-based education, and deliberate practice [1,2]. Educational tools such as quiz-based platforms, virtual reality (VR), simulation, escape rooms, serious games, and mobile learning applications are increasingly incorporated into medical curricula to promote learner engagement, reinforce knowledge acquisition, strengthen teamwork, and provide psychologically safe opportunities for learners to practice clinical decision-making before caring for patients [3-6]. Emerging evidence suggests that well-designed gaming interventions can improve learner engagement, short-term knowledge retention, teamwork, and procedural competence. Simulation-based mastery learning (SBML), in particular, has demonstrated measurable improvements in clinical performance and selected patient outcomes when paired with structured feedback and repeated practice. In medical education, the appeal is clear: interactive challenges, immediate feedback, and psychologically safe rehearsal of difficult scenarios. Yet the “game turn” also raises concerns about superficial learning, inequities, and subtle shifts in professional identity. In a study by Cook et al., a balanced, evidence-attentive view suggests gaming can add real value, especially when aligned with competency-based goals and followed by structured debriefing, but it is not a cure-all, and poor design can backfire [6,7].

As gaming and gamification continue to evolve within health professions education, a critical examination of their educational foundations, current applications, and evidence base is needed to guide effective implementation and inform future curricular innovation.

This narrative review examines the evolving role of gaming and gamification across the continuum of medical education, from undergraduate medical education to residency training and organizational learning. We first describe the approach used to identify and select the literature included in this review. Next, we discuss the educational theories that provide the foundation for game-based learning, followed by an examination of how gaming is currently implemented in pre-clerkship education, clinical training, residency programs, and institutional initiatives. We then synthesize the evidence regarding the educational effectiveness of gaming and gamification, highlighting both demonstrated benefits and current limitations. Finally, we examine practical considerations for successful implementation, discuss emerging trends and future directions, and offer recommendations for educators seeking to integrate gaming into competency-based medical education while maintaining professionalism, equity, and patient-centered learning.

## Review

Study selection approach

A targeted literature search was conducted to identify contemporary evidence on gaming and gamification in medical education. PubMed, ERIC, and Google Scholar searches were conducted to identify relevant publications published between 2005 and May 2026. Search terms included gamification, game-based learning, serious games, gaming, medical education, health professions education, simulation, VR, escape rooms, residency education, competency-based medical education, and technology-enhanced learning. Additional articles were identified through manual searches of reference lists from relevant publications.

Priority was given to systematic reviews, meta-analyses, randomized controlled studies, multicenter educational research, and seminal publications that have significantly influenced the field of game-based learning in health professions education [4-6,8,9]. Additionally, studies describing innovative educational interventions, including the use of Kahoot, Anki, escape rooms, SBML, and VR, were included to illustrate practical applications across undergraduate medical education, residency training, and organizational learning [3,10-17].

A narrative review methodology was selected based on its relevance to the objectives of the manuscript. We evaluate the evidence supporting educational effectiveness, identify potential limitations and ethical considerations, and highlight evidence-informed recommendations for successful implementation across the medical education continuum.

Peer-led game-based learning in pre-clerkship education

The pre-clerkship phase of medical education has become one of the most active environments for implementing gaming and gamification strategies. As medical curricula continue to shift toward active, learner-centered, and competency-based education, educators have increasingly adopted game-based approaches to supplement traditional lectures and laboratory instruction. These strategies are designed not only to increase learner engagement but also to promote active participation, collaboration, and the application of foundational scientific knowledge in meaningful educational contexts [4,6,8]. Student-led learning originates from the collective use of game-based platforms such as Kahoot/Quizizz tournaments, puzzle-based “escape” sessions, and flashcard sprints around Anki decks. Small studies and reviews in medicine and allied fields report improved engagement and short-term knowledge measures when platforms like Kahoot are used for formative assessment. Kahoot is commonly used during lectures and review sessions to provide immediate formative feedback while encouraging participation in a low-stakes learning environment. Studies conducted in anatomy, histology, and immunology courses have demonstrated that these platforms improve learner engagement, classroom participation, and short-term knowledge acquisition while creating a more interactive educational experience [3,10,18,19]. However, systematic reviews suggest that improvements in examination performance are generally modest and depend largely on the quality of instructional design rather than the technology itself [4,8].

Another widely adopted strategy, spaced repetition via Anki, has been associated with higher exam performance among medical students in cohort studies [8]. Unlike competitive gaming platforms, Anki emphasizes self-directed learning and repeated knowledge retrieval, both of which have been associated with improved academic performance and preparation for licensing examinations [11]. Meanwhile, escape-room formats, often designed by students or educators, have been deployed in anatomy and therapeutics and are generally well-liked while showing pre/post gains [19-22]. These examples illustrate a broader pattern: peer communities iteratively remix content into games to make heavy material feel doable and social.

Practical applications

Across many campuses, student organizations host game-based review sessions, trivia competitions, and specialty-themed challenges to reinforce course content before examinations (e.g., cardiology “boss battles”). Educational escape rooms are frequently incorporated into patient safety training, Advanced Cardiovascular Life Support (ACLS) review sessions, and interprofessional learning activities, while anatomy societies and student interest groups develop puzzle-based exercises and diagnostic case challenges to promote collaborative problem-solving. Many pre-clerkship course societies set up puzzle stations; interest groups run “diagnostic hunt” quests; and phase 1 courses integrate quiz-show segments at the end of lectures. Reports from pharmacy and medical programs document such activities as drivers of attendance and group cohesion, which collectively foster learner engagement, strengthen collaboration, and create supportive learning environments that encourage active participation rather than passive knowledge acquisition [19,21,22].

What the evidence says

Growing evidence suggests that gaming and gamification can improve learning in medical education through enhanced learner engagement, motivation, and short-term knowledge acquisition. However, Sailer and Cook [4,6,8] argue that these benefits are modest and highly dependent on the instructional design used on the gaming platforms. Systematic reviews and meta-analyses demonstrate that game-based learning improves learner engagement and behavioral outcomes [23], but note that the overall quality of evidence remains moderate because many studies use small samples, single institutions, and short-term outcome measures.

Stronger evidence exists for simulation-based learning, which shares many characteristics with serious games. Technology-enhanced simulation has been shown to produce large improvements in learners' knowledge and clinical skills, as well as moderate improvements in patient outcomes when compared with no educational intervention [7]. More recent narrative reviews further suggest that emerging technologies, including web-based games, virtual simulations, and social media-enhanced learning environments, offer promising opportunities to increase learner engagement. Nevertheless, successful implementation requires careful attention to educational design, resource availability, and integration with evidence-based teaching strategies rather than reliance on technology alone [19,24,25].

Risks

Despite its potential, gamification carries important limitations. Poorly designed gamified learning environments can shift learners' focus from reflective practice and clinical reasoning toward the pursuit of external rewards, such as badges, points, and leaderboard rankings [8]. In a longitudinal study conducted outside of medical education, Hanus and colleagues [8] reported that students in a gamified course experienced lower motivation, satisfaction, and perceived empowerment over time compared with those in a non-gamified control group. A meta-analytic review by Sailer et al. further suggests that while gamification consistently supports cognitive learning outcomes, its effects on motivation and behavior are more variable and appear to depend on thoughtful instructional design, particularly the balance between collaborative and competitive elements [10].

In high-stakes professional training, competitive features such as leaderboards may inadvertently increase anxiety, shame, or disengagement among lower-performing learners while disadvantaging students who are less familiar or comfortable with game-based environments. Equity also remains a significant concern, as high-fidelity VR simulations and commercial gamification platforms require substantial financial and technological resources, potentially limiting access and widening educational disparities [12]. From an ethical perspective, critics argue that framing life-and-death clinical decision-making as a game risks trivializing the seriousness of patient care and oversimplifying the relational, contextual, and moral complexities that characterize professional practice [20].

Organizational gamification: engagement at scale

Beyond classrooms, programs and institutions are experimenting with game elements to lift engagement and safety behaviors. Examples from MedEdPORTAL curricula include patient-safety escape rooms to surface hazards and increase familiarity with reporting systems [15], intern “social capital” escape rooms to accelerate team cohesion, and gamified hand-hygiene platforms that pair feedback with rewards [6,14,26]. While results vary, multiple reports suggest these interventions can raise participation and awareness when embedded in broader quality improvement strategies; importantly, they are add-ons rather than replacements for policy, supervision, and audit-and-feedback [26].

Gaming in graduate medical education

Many residency programs run “Jeopardy”-style board reviews, case-race mornings, and escape rooms targeting teamwork, ACLS/PALS, or patient-safety workflows [14,16]. Specialty examples include nephrology-themed escape rooms to attract internal medicine residents and emergency medicine didactic escape games for diagnostic teamwork [27]. Although SBML is not a gaming intervention, it is included here because many gamified educational activities in residency are implemented within simulation-based environments and share key educational principles, including deliberate practice, structured feedback, repetition, and competency-based progression. Evidence from SBML has demonstrated reductions in real-world procedural complications and lower catheter-associated bloodstream infection rates following central line training [11], illustrating the potential impact of well-designed, simulation-based educational strategies on clinical practice. Similarly, VR simulation has been shown to improve procedural performance metrics in endoscopy training [28]. These residency-level applications illustrate how game-based learning can enhance simulation-based education when paired with mastery learning, structured assessment, and debriefing, supporting competencies that may ultimately contribute to improved clinical practice.

What best practice looks like

Studies have consistently demonstrated (see Table 1) that meaningful learning occurs when game-based activities are intentionally integrated into the curriculum and aligned with clearly defined learning objectives [4,5,8]. Conversely, poorly designed interventions that prioritize competition, entertainment, or extrinsic rewards over educational outcomes may produce only modest learning gains and can inadvertently undermine learner motivation and engagement [7,8]. As medical educators increasingly adopt gaming across various levels of medical education, residency training, and continuing professional development, careful attention must be given to designing activities that promote competency development, reflective practice, teamwork, and professional identity formation.

**Table 1:** Evidence-informed best practices for implementing gaming and gamification in medical education

Best Practice	Educational Rationale	Practical Application	Supporting Evidence
Align gaming with competency-based learning objectives	Game-based activities should reinforce curricular outcomes, entrustable professional activities (EPAs), milestones, and licensing examination objectives rather than isolated factual recall. Alignment ensures that gaming supports competency development and authentic clinical practice.	Design games around clinical reasoning, communication, teamwork, ethical decision-making, and procedural competencies rather than trivia or recall-based questions.	Simulation-based learning is most effective when integrated into curricula with clearly defined learning objectives and competency-based assessment [4,5,8,9].
Incorporate deliberate practice and timely feedback	Repeated practice with immediate feedback promotes mastery learning, allowing learners to identify deficiencies and progressively improve performance before advancing.	Include iterative gameplay, faculty or peer feedback, and minimum passing standards (MPS) that require learners to demonstrate competence before progressing.	Simulation-based mastery learning improves procedural competence, learner confidence, and patient safety outcomes, including reductions in catheter-related bloodstream infections [5,9,13,15,16,20].
Facilitate structured debriefing and reflection	Reflection transforms game experiences into meaningful learning by encouraging learners to connect decisions made during gameplay with clinical reasoning, communication, ethics, and patient care.	Conduct structured faculty-led debriefing sessions following simulations, escape rooms, or team-based games to reinforce learning and identify opportunities for improvement.	Debriefing enhances clinical reasoning, knowledge retention, reflective practice, and professional identity formation [5,9,18,24].
Promote inclusive and collaborative learning environments	Collaboration, psychological safety, and equitable participation foster deeper learning than highly competitive environments. Excessive competition may increase anxiety and reduce learner engagement.	Use cooperative challenges, rotating team roles, peer teaching, and multiple opportunities for participation while limiting excessive reliance on leaderboards and rankings.	

Utilizing current evidence, several characteristics consistently emerge as essential for effective implementation. These include aligning gaming activities with competency-based outcomes, incorporating deliberate practice and timely feedback, facilitating structured debriefing and reflection, and fostering inclusive, collaborative learning environments that support meaningful participation for all learners [5,9,15,23].

Alignment With Competencies

Game tasks map to entrustable professional activities (EPAs) or exam blueprints, not just “fun facts” [9]. The effectiveness of game-based learning depends on its alignment with clearly defined educational objectives rather than the novelty of the gaming platform. Successful gaming interventions are intentionally designed with clearly defined objectives to reinforce competency-based medical education by mapping activities to curricular outcomes, EPAs, milestone achievement, or licensing examination blueprints [9]. Rather than rewarding isolated facts or rapid recall, educational games should promote higher-order skills such as clinical reasoning, communication, teamwork, and ethical decision-making. Grounded in educational theories such as Bloom's taxonomy, constructivism, and experiential learning, this approach encourages learners to apply, analyze, evaluate, and integrate knowledge in authentic clinical contexts, thereby supporting meaningful learning and transfer to practice.

Research on simulation-based education consistently demonstrates that learners achieve greater educational gains when activities are integrated into the curriculum and aligned with explicit learning objectives, allowing educators to assess competency development while reinforcing authentic clinical practice [5,9]. This alignment ensures that gaming serves as an instructional strategy that supports professional identity formation and clinical competence, rather than functioning solely as an engaging educational activity or assessment tool [4,8].

Deliberate Practice + Feedback

Iterative trials, expert feedback, and minimum passing standards (MPS) prevent hollow progress [7,15]. Deliberate practice is a cornerstone of effective game-based learning because it emphasizes repeated performance, immediate feedback, and progressive skill development until learners achieve predefined standards of competence. Simulation-based mastery learning exemplifies this approach by requiring learners to demonstrate proficiency before progressing to patient care, thereby reinforcing both technical and clinical decision-making skills [5,9]. Constructive critical feedback from faculty or peers enables learners to identify performance gaps, correct errors, and refine their approach through repeated practice, resulting in deeper learning than one-time educational encounters [13,15].

Rather than rewarding completion alone, effective gaming interventions establish MPS that emphasize mastery and continuous improvement over competition. For example, a student does not earn credit for completing a virtual central line simulation; instead, each critical step must be correctly performed before progressing. Feedback should then be given if the standards have not been met, and the activity should be repeated until competency is achieved. This approach has been associated with improvements in procedural competence, learner confidence, and patient safety outcomes, including reductions in catheter-related bloodstream infections following simulation-based central venous catheter training [13,16,20].

Debriefing and Reflection

Structured debriefs connect game actions to patient care, ethics, and team dynamics [18,24]. Debriefing is an essential component of effective game-based learning because it transforms game experiences into meaningful educational opportunities. Structured reflection encourages learners to critically examine their decisions, connect gameplay to clinical reasoning, and explore the ethical, professional, and interpersonal dimensions of patient care [5,9]. Following simulation exercises, escape rooms, or team-based games, facilitated debriefing allows learners to discuss diagnostic reasoning, communication strategies, teamwork, and patient safety while identifying areas for future improvement. This reflective process promotes deeper learning by linking game-based experiences to authentic clinical practice rather than viewing games as isolated educational activities [18]. Emerging evidence also suggests that guided reflection strengthens knowledge retention and professional identity formation by encouraging learners to consider how their decisions influence patient outcomes and collaborative team performance [24]. Consequently, debriefing should be viewed as an integral component of game-based learning rather than an optional post-activity discussion.

Inclusive, Collaborative Mechanics

Co-op challenges and role rotation avoid demoralizing rank lists and widen participation [23]. Evidence suggests that game-based learning is most effective when it promotes collaboration, psychological safety, and equitable participation among learners. Although competitive elements such as points and leaderboards may increase engagement for some students, excessive competition can discourage participation, heighten anxiety, and shift learners' attention from mastery to performance [7,8]. Inclusive game design also recognizes that learners differ in their previous gaming experience, technological access, and learning preferences. Designing various ways to participate and valuing diverse contributions creates a more supportive learning environment and reduces barriers to engagement [23]. By prioritizing collaboration over competition, educators can foster teamwork, inclusivity, and professional behaviors while ensuring that gaming enhances learning for all students rather than only those who thrive in highly competitive environments.

Peer Learning and Collaborative Knowledge Construction 

Peer-led games and puzzles are increasingly common in pre-clerkship because they promote learner engagement and collaborative learning and create social accountability while offering bite-sized wins during heavy content weeks. While these approaches are effective, evidence suggests that “only via gaming” risks narrowing learning to fast-recall performance. Opportunities to promote higher-order clinical reasoning, professional judgment, and sustained behavioral change may also be lost. Systematic reviews indicate that the greatest educational benefits are achieved when game-based activities are complemented by authentic clinical scenarios, structured debriefing, meaningful assessment, and reflective practice [5,9].

## Conclusions

Gaming in medical education is most powerful as a method, not a mandate. Used thoughtfully, it enhances learner engagement, knowledge acquisition, collaboration, and clinical skill development across the continuum of medical education. Evidence suggests that its effectiveness depends not only on the technology itself but also on thoughtful instructional design, alignment with competency-based learning objectives, deliberate practice, timely feedback, and structured reflection. Therefore, the task for educators is not to ask, “game or no game,” but to engineer learning: align mechanics to competencies, privilege feedback over badges, and pair play with reflection. When thoughtfully designed and grounded in sound educational principles, gaming becomes more than an engaging instructional innovation; it becomes a powerful catalyst for developing competent, collaborative, and compassionate physicians prepared to meet the evolving demands of modern healthcare.
